# Crystal structure of *cis*-diamminebis(nitrito-κ*N*)platinum(II)

**DOI:** 10.1107/S2056989015004879

**Published:** 2015-03-14

**Authors:** Volker Kahlenberg, Thomas Gelbrich, Richard Tessadri, Frederik Klauser

**Affiliations:** a University of Innsbruck, Institute of Mineralogy & Petrography, Innrain 52, A-6020 Innsbruck, Austria; b University of Innsbruck, Institute of Pharmacy, Innrain 80, 6020 Innsbruck, Austria; c MED-EL Medical Electronics, Fürstenweg 77a, A-6020 Innsbruck, Austria

**Keywords:** crystal structure, hydrogen bonding, topology, platinum(II), *cis*-isomer

## Abstract

Single crystals of *cis*-[Pt(NO_2_)_2_(NH_3_)_2_], were obtained by means of hyper­saturation directly out of a plating electrolyte. The square-planar coordination environment of the divalent Pt^II^ atom is formed by four N atoms belonging to two ammine and two monodentate nitrite ligands. The ligands adopt a *cis* configuration. The crystal structure contains stacks of close-packed mol­ecules which run parallel to [001]. There are nine crystallographically independent inter­molecular N—H⋯O hydrogen bonds, resulting in a hydrogen-bonded **hxl**-type framework in which each mol­ecule serves as an eight-connected node. Four of the nine distinct hydrogen bonds connect complexes which belong to the same close-packed column parallel to [001]. In contrast to the previously reported crystal structure of the *trans* isomer, the title structure does not display intra­molecular hydrogen bonding.

## Abstract 2

The Pt^II^ atoms are coordinated in a square-planar fashion. Extensive N—H⋯O bonding between neighbouring mol­ecules results in an hydrogen-bonded framework which has the **hxl** topology.

## Chemical context   

Several platinum salt systems have been studied intensively for the electrodeposition of platinum and platinum alloys with regard to their economic availability and their hydrolysis behaviour in solution. An excellent summary of the different systems can be found in the review paper of Baumgärtner & Raub (1988 ▸). One of the materials that has frequently been used as a platinum source in electrochemical deposition processes is diamminebis(nitrito)platinum(II), better known as platinum p-salt. Aqueous slurries of this compound are especially suited for the production of dense and homogeneous coatings. Indeed, this material was used for electroplating by Keitel & Zschiegner as early as 1931 ▸. The ligands stabilize the platinum ion in solution and prevent the oxidation of Pt^II^ to Pt^IV^. However, to enable electrochemical platinum deposition out of this stable complex, temperatures of approximately 363 K are required.

## Structural commentary   

**Figure 1:**
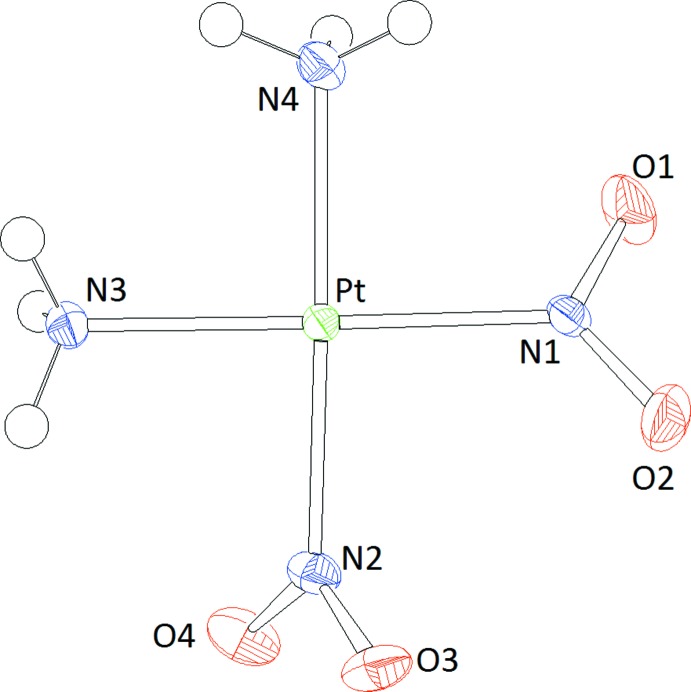
Representation of the mol­ecular structure of *cis*-[Pt(NH_3_)_2_(NO_2_)_2_]. Displacement ellipsoids are drawn at the 60% probability level.

The asymmetric unit of the title structure contains a neutral [Pt(NH_3_)_2_(NO_2_)_2_] complex whose ammine and nitrito ligands adopt a *cis* configuration (Fig. 1 ▸). The Pt^II^ atom is coordinated by one nitro­gen atom from each of the four ligands in a square-planar fashion. The distances between the positions of Pt, N1, N2, N3 and N4 and the corresponding least-squares plane are −0.0018 (13), −0.0191 (15), 0.0202 (16), −0.0192 (15) and 0.0199 (15) Å, respectively. As expected, the Pt—N bonds to the ammine ligands, 2.039 (3) and 2.052 (3) Å, are somewhat longer than the Pt—N bonds to each of the monodentate nitrite groups, 1.995 (3) and 2.001 (4) Å. The largest deviation of any N—Pt—N bond angle from its ideal value is observed in the angle between the two nitrite groups [N1—Pt—N2 = 93.06 (13)°]. The bond-valence sum for the four cation–anion inter­actions around the Pt^II^ atom is 2.256 valence units according to a calculation using the parameter set for the Pt—N bond given by Brown (2002 ▸). The NO_2_ planes defined by the two nitrite groups form angles of 38.6 (2) and 61.6 (2)°, respectively, with the least-squares plane of the central PtN_4_ unit. Moreover, these NO_2_ planes are twisted against one another by 62.4 (4)°.

## Supra­molecular features   

**Figure 2:**
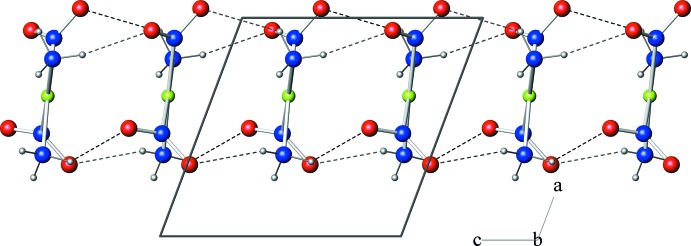
Projection perpendicular to (001), showing a single column of *cis*-[Pt(NH_3_)_2_(NO_2_)_2_] mol­ecules which propagate parallel to [001]. Pt (green), N (blue), O (red) and H (grey) atoms are drawn as spheres. Dashed lines indicate hydrogen bonds.

Mol­ecules are arranged into columns propagating parallel to [001] in such a way that neighbouring *cis*-[Pt(NH_3_)_2_(NO_2_)_2_] units are related by glide mirror symmetry, and their central PtN_4_ planes form an angle of approximately 85° with the stacking vector (Fig. 2 ▸). The metal coordination centres of neighbouring mol­ecules in the resulting stack are separated by 3.5486 (2) Å and the corresponding inter­molecular Pt⋯Pt⋯Pt angle is 176.1°. By comparison, the double value of the default Pt contact radius (Bondi, 1964 ▸) is 3.44 Å. The distances between platinum ions belonging to neighbouring columns are considerably longer and correspond to the length of the *a* axis [6.8656 (5) Å].

**Figure 3:**
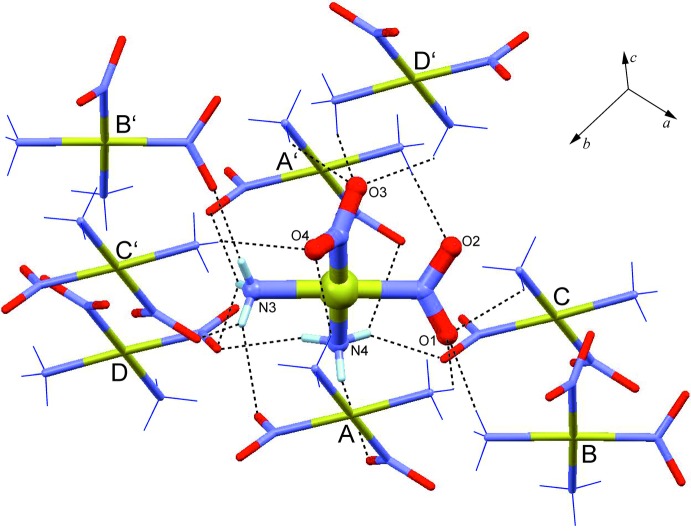
N—H⋯O hydrogen-bonding inter­actions (dashed lines) in *cis*-[Pt(NH_3_)_2_(NO_2_)_2_] between a central mol­ecule and eight neighbouring mol­ecules, denoted A–D and A′–D′. [Symmetry operations used to generate equivalent mol­ecules: (A) *x*, −*y* + 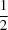, *z* − 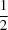; (B) *x* − 1, *y*, *z*; (C) *x* + 1, *y* + 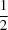, *z* + 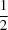; (D) −*x* + 1, *y* + 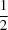, −*z* + 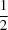; (A′) *x*, −*y* + 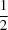, *z* + 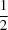; (B′) *x* − 1, *y*, *z*; (C′) *x* − 1, −*y* + 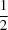, *z* − 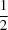; (D′) −*x* + 1, *y* − 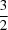, −*z* + 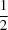.]

All six available hydrogen-bonding donor sites of the ammine groups and each of the four nitrite O atoms are engaged in nine inter­molecular N—H⋯O bonds (Table 1 ▸; Fig. 3 ▸), whose H⋯O distances lie between 2.14 and 2.57 Å. Four of these inter­actions are formed within the same supra­molecular stack parallel to [001], *i.e.*, neighbouring mol­ecules within this one-periodic structure are connected to one another by four-point N—H⋯O connections.

**Figure 4:**
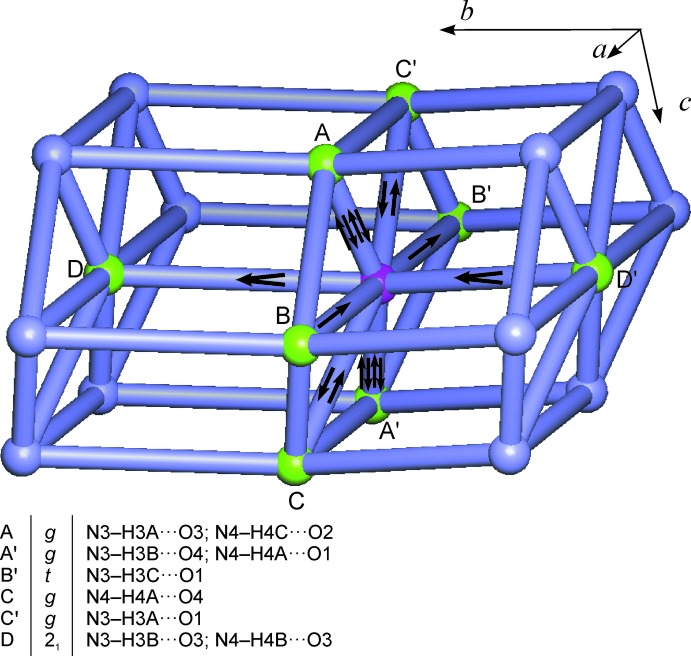
The N—H⋯O hydrogen-bonded F18_8_[3^6^.4^18^.5^3^.6-**hxl**] structure of *cis*-[Pt(NH_3_)_2_(NO_2_)_2_]. Mol­ecules are represented as nodes and their hydrogen-bonding connections as links between them. Individual N—H⋯O inter­actions between a central mol­ecule (magenta) and eight neighbouring mol­ecules (A–D and A′–D′; green) are indicated by arrows (H→O). The inter­actions between the central mol­ecule and mol­ecules A and A′ correspond to the columnar arrangement shown in Fig. 2 ▸. The hydrogen bonds donated by the central mol­ecule and the symbols for the symmetry operations associated with them are given at the bottom. For symmetry codes, see caption to Fig. 3 ▸.

In total, each mol­ecule is engaged in 18 hydrogen-bonding inter­actions which link it to eight neighbours *via* two four-point, four two-point and two one-point connections. The resulting N—H⋯O-bonded framework structure has the topology of the hexa­gonal lattice (**hxl**) (O’Keeffe *et al.*, 2008 ▸). Fig. 4 ▸ gives a graphical representation of this hydrogen-bonding structure (HBS) in the style proposed by Hursthouse *et al.* (2015 ▸). It shows that 14 out of the 18 hydrogen-bonding inter­actions of an individual mol­ecule lie within the (010) planes, and eight of these within the same column parallel to [001] (all inter­actions involving the central mol­ecule and either mol­ecule of A and A′). The descriptor of this HBS is F18_8_[3^6^.4^18^.5^3^.6-**hxl**] according to the methodology proposed by Hursthouse *et al.* (2015 ▸). Additionally, the sequence [*g* ^IV^.*t.g* ^II^.*g* ^IV^.*t.g* ^II^.2_1_^II^.2_1_^II^] describes the symmetry operations and numbers of hydrogen bonds involved in the eight distinct connections between two mol­ecules which define this HBS.

## Database survey   

Various platinum(II) complexes, including diamminebis(nitrito)platinum(II), have been studied intensively as precious metal sources in electrochemical deposition processes (Keitel & Zschiegner, 1931 ▸; Baumgärtner & Raub, 1988 ▸). Previous reports by Khranenko *et al.* (2007 ▸), Laligant *et al.* (1991 ▸) and Madarász *et al.* (2009 ▸) contain crystal structures with a close relationship to *cis*-[Pt(NH_3_)_2_(NO_2_)_2_,] and of these the *trans* isomer and its Pd analogue (Madarász *et al.*, 2009 ▸) are of particular inter­est.

**Figure 5:**
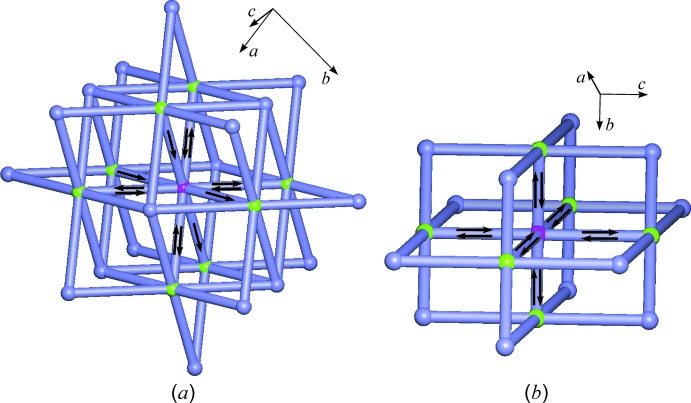
N—H⋯O hydrogen-bonded frameworks formed by structural analogues of the title compound: (*a*) F12_8_[4^24^.6^4^-**bcu**] structure of *trans*-[Pt(NH_3_)_2_(NO_2_)_2_] and (*b*) F12_6_[4^12^.6^3^-**pcu**] structure of *trans*-[Pd(NH_3_)_2_(NO_2_)_2_]_._ Mol­ecules are represented as nodes and hydrogen-bonded connections as the links between them. Individual N—H⋯O inter­actions between a central mol­ecule (magenta) and eight neighbouring mol­ecules (A–D, A′–D′; green) are indicated by arrows (H→O).

In the crystal structure of *trans*-[Pt(NH_3_)_2_(NO_2_)_2_], the Pt^II^ atom is coordinated in a square-planar fashion by N atoms of the four ligands. The shortest Pt⋯Pt distance is much longer (4.84 Å) than in the title structure as there are no close-packed columnar units similar to those found in the *cis* analogue (Fig. 2 ▸). In addition to intra­molecular N—H⋯O inter­actions, each of the six ammine hydrogen atoms of the *trans*-Pt(NH_3_)_2_(NO_2_)_2_ mol­ecule is employed in just one inter­molecular N—H⋯O inter­action in such a way that each mol­ecule is hydrogen-bonded to eight neighbouring mol­ecules. Altogether, an individual *trans*-[Pt(NH_3_)_2_(NO_2_)_2_] mol­ecule is engaged in twelve hydrogen-bonding inter­actions which are grouped into four two-point and four one-point connections (Fig. 5 ▸ *a*). The underlying net of the resulting HBS has the body-centered cubic (**bcu**) topology (O’Keeffe *et al.*, 2008 ▸) and the descriptor according to Hursthouse *et al.* (2015 ▸) for this HBS is F12_8_[4^24^.6^4^-**bcu**].

The structure of the palladium analogue, *trans*-[Pd(NH_3_)_2_(NO_2_)_2_], also displays a square-planar metal coordination by four N atoms of the ammine and nitrite ligands, and the shortest inter­molecular Pd⋯Pd distance is 5.42 Å. As in the *trans*-Pt analogue, each H atom is employed in just one inter­molecular N—H⋯O bond so that each mol­ecule is engaged in twelve individual hydrogen-bonding inter­actions. In contrast to the *trans*-Pt^II^ analogue, these are exclusively two-point anti­parallel contacts to just six neighbours (Fig. 5 ▸ *b*). The underlying net of the 3-periodic HBS formed as a result of these inter­actions, has the primitive cubic (**pcu**) topology (O’Keeffe *et al.*, 2008 ▸) and its descriptor is F12_6_[4^12^.6^3^-**pcu**].

The HBSs formed by three structural analogues (Figs. 4 ▸ and 5 ▸) are each based on inter­molecular N—H⋯O inter­actions involving the same set of six hydrogen-bonding donor and four hydrogen-bonding acceptor sites per mol­ecule. However, the ensuing extensive hydrogen bonding results in three different framework structures, each of which was found to possess the topology of a particular Bravais lattice.

## Synthesis and crystallization   

Single crystals of *cis*-Pt(NH_3_)_2_(NO_2_)_2_ were obtained by means of hypersaturation directly out of a plating electrolyte. In order to grow larger single crystals, the water from the solution was partly evaporated at ambient temperature over a time span of two months. For structure analysis, a single crystal of good optical quality showing sharp extinction when imaged between crossed polarizers was selected and mounted on the tip of a 0.025 mm thick Mylar cryoloop (LithoLoops, Mol­ecular Dimensions Inc.) using a perfluoro­polyether inert oil (Hampton Research). Subsequently, the crystal was flash-cooled in a 173 (2) K dried air stream generated by an Oxford Cryosystems Desktop Cooler. A preliminary unit cell determination using on Oxford Diffraction Gemini Ultra single crystal diffractometer resulted in a set of lattice parameters that could not be found in the recent WEB based version of the Inorganic Crystal Structure Database (ICSD, 2014 ▸). Therefore, we decided to perform a full data collection for structure solution.

## Refinement   

Crystal data, data collection and structure refinement details are summarized in Table 2 ▸. A data set corresponding to a hemisphere of reciprocal space was collected. Structure solution by direct methods revealed the positions of all non-hydrogen atoms. All missing hydrogen atoms were identified from difference Fourier calculations. The H atoms of NH_3_ groups were idealized and included as rigid groups allowed to rotate but not tip (N—H = 0.91 Å), with their displacement parameters set to *U*_iso_(H) = 1.5*U*_eq_(N) of the parent N atom. The largest peaks of the final difference electron density map were close to the position of the metal atom.

## Analysis of hydrogen-bonded structures   

The topologies of HBSs were determined and classified with the programs *ADS* and *IsoTest* of the *TOPOS* package (Blatov, 2006 ▸) in the manner described by Baburin & Blatov (2007 ▸). The topology graphs for HBSs (Figs. 4 ▸ and 5 ▸) are based on nets drawn with the *IsoCryst* program of the *TOPOS* package. The HBS of the title structure was defined from nine N—H⋯O inter­actions, which are listed in Table 1 ▸. Not included in this analysis was the inter­action N4—H4*C*⋯O3(*x*, −*y* + 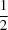, *z* − 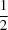) (H⋯*A* = 2.63 Å), inter­preted as an additional opportunistic contact between the central mol­ecule and mol­ecule A. The definition of the HBSs of *trans*-[Pt(NH_3_)_2_(NO_2_)_2_] and *trans*-[Pd(NH_3_)_2_(NO_2_)_2_] were based on the inter­molecular N—H⋯O inter­actions listed in Tables S1 and S2, respectively, of the Supporting information.

## Supplementary Material

Crystal structure: contains datablock(s) I. DOI: 10.1107/S2056989015004879/wm5133sup1.cif

Structure factors: contains datablock(s) I. DOI: 10.1107/S2056989015004879/wm5133Isup2.hkl

Supporting information file. DOI: 10.1107/S2056989015004879/wm5133Isup3.pdf

CCDC reference: 1053034

Additional supporting information:  crystallographic information; 3D view; checkCIF report

## Figures and Tables

**Table 1:** Hydrogen-bond geometry (, )

*D*H*A*	*D*H	H*A*	*D* *A*	*D*H*A*
N3H3*A*O3^i^	0.91	2.27	3.026(5)	140
N3H3*A*O1^ii^	0.91	2.49	3.107(5)	126
N3H3*B*O4^iii^	0.91	2.22	2.941(5)	136
N3H3*C*O1^iv^	0.91	2.12	3.015(5)	169
N3H3*B*O3^v^	0.91	2.30	2.976(5)	131
N4H4*A*O4^vi^	0.91	2.56	3.392(4)	153
N4H4*A*O1^iii^	0.91	2.57	3.261(5)	133
N4H4*B*O3^v^	0.91	2.14	2.994(4)	156
N4H4*C*O2^i^	0.91	2.18	3.072(5)	167

Symmetry codes: (i) 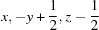; (ii) 
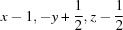
; (iii) 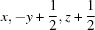; (iv) 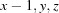; (v) 
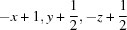
; (vi) 
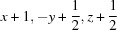
.

**Table 2:** Experimental details

Crystal data	
Chemical formula	[Pt(NO_2_)_2_(NH_3_)_2_]
*M*_r_	321.18
Crystal system, space group	Monoclinic, *P*2_1_/*c*
Temperature (K)	173
*a*, *b*, *c* ()	6.8656(5), 12.6428(8), 7.0931(5)
()	110.579(8)
*V* (^3^)	576.40(7)
*Z*	4
Radiation type	Mo *K*
(mm^1^)	24.30
Crystal size (mm)	0.20 0.12 0.02

Data collection	
Diffractometer	Agilent Xcalibur (Ruby, Gemini ultra)
Absorption correction	Analytical [*CrysAlis PRO* (Agilent, 2014 ▸), based on expressions derived by Clark Reid (1995 ▸)]
*T*_min_, *T*_max_	0.036, 0.609
No. of measured, independent and observed [*I* > 2(*I*)] reflections	3435, 1061, 972
*R*_int_	0.031
(sin /)_max_ (^1^)	0.602

Refinement	
*R*[*F* ^2^ > 2(*F* ^2^)], *wR*(*F* ^2^), *S*	0.018, 0.044, 1.05
No. of reflections	1061
No. of parameters	85
H-atom treatment	H-atom parameters constrained
_max_, _min_ (e ^3^)	1.02, 0.92

Computer programs: *CrysAlis PRO* (Agilent, 2014 ▸), *SIR2002* (Burla *et al.*, 2003 ▸), *SHELXL2014* (Sheldrick, 2015 ▸), *ATOMS for Windows* (Dowty, 2011 ▸), *ORTEP-3 for Windows* and *WinGX* (Farrugia, 2012 ▸), *Mercury* (Macrae *et al.*, 2006 ▸), *TOPOS* (Blatov, 2006 ▸), *PLATON* (Spek, 2009 ▸) and *publCIF* (Westrip, 2010 ▸).

## supplementary crystallographic information

### Crystal data

**Table:**

[Pt(NO_2_)_2_(NH_3_)_2_]	*F*(000) = 576
*M**_r_* = 321.18	*D*_x_ = 3.701 Mg m^−^^3^
Monoclinic, *P*2_1_/*c*	Mo *K*α radiation, λ = 0.71073 Å
*a* = 6.8656 (5) Å	Cell parameters from 2142 reflections
*b* = 12.6428 (8) Å	θ = 3.5–28.5°
*c* = 7.0931 (5) Å	µ = 24.30 mm^−^^1^
β = 110.579 (8)°	*T* = 173 K
*V* = 576.40 (7) Å^3^	Thin plate, yellow
*Z* = 4	0.20 × 0.12 × 0.02 mm

### Data collection

**Table:**

Agilent Xcalibur (Ruby, Gemini ultra) diffractometer	1061 independent reflections
Radiation source: sealed tube	972 reflections with *I* > 2σ(*I*)
Graphite monochromator	*R*_int_ = 0.031
Detector resolution: 10.3575 pixels mm^-1^	θ_max_ = 25.4°, θ_min_ = 3.5°
ω scans	*h* = −8→7
Absorption correction: analytical [*CrysAlis PRO* (Agilent, 2014), based on expressions derived by Clark & Reid (1995)]	*k* = −11→15
*T*_min_ = 0.036, *T*_max_ = 0.609	*l* = −6→8
3435 measured reflections	

### Refinement

**Table:**

Refinement on *F*^2^	Secondary atom site location: difference Fourier map
Least-squares matrix: full	Hydrogen site location: inferred from neighbouring sites
*R*[*F*^2^ > 2σ(*F*^2^)] = 0.018	H-atom parameters constrained
*wR*(*F*^2^) = 0.044	*w* = 1/[σ^2^(*F*_o_^2^) + (0.0222*P*)^2^] where *P* = (*F*_o_^2^ + 2*F*_c_^2^)/3
*S* = 1.05	(Δ/σ)_max_ = 0.002
1061 reflections	Δρ_max_ = 1.02 e Å^−^^3^
85 parameters	Δρ_min_ = −0.92 e Å^−^^3^
0 restraints	Extinction correction: *SHELXL2014* (Sheldrick, 2015), Fc^*^=kFc[1+0.001xFc^2^λ^3^/sin(2θ)]^-1/4^
Primary atom site location: structure-invariant direct methods	Extinction coefficient: 0.0024 (2)

### Special details

**Table:**

Experimental. Absorption correction: CrysAlis PRO (Agilent, 2014) Analytical numeric absorption correction using a multifaceted crystal model based on expressions derived by Clark & Reid (1995)
Geometry. All e.s.d.'s (except the e.s.d. in the dihedral angle between two l.s. planes) are estimated using the full covariance matrix. The cell e.s.d.'s are taken into account individually in the estimation of e.s.d.'s in distances, angles and torsion angles; correlations between e.s.d.'s in cell parameters are only used when they are defined by crystal symmetry. An approximate (isotropic) treatment of cell e.s.d.'s is used for estimating e.s.d.'s involving l.s. planes.

### Fractional atomic coordinates and isotropic or equivalent isotropic displacement parameters (Å^2^)

**Table:**

	*x*	*y*	*z*	*U*_iso_*/*U*_eq_	
Pt	0.64201 (3)	0.24522 (2)	0.18480 (2)	0.00838 (12)	
O2	0.9425 (5)	0.0838 (2)	0.3550 (5)	0.0214 (7)	
O1	1.0428 (4)	0.1964 (3)	0.1858 (4)	0.0234 (7)	
O3	0.4941 (4)	0.0546 (2)	0.2976 (4)	0.0182 (7)	
O4	0.3278 (4)	0.0944 (3)	−0.0112 (4)	0.0217 (7)	
N1	0.9066 (5)	0.1640 (3)	0.2494 (5)	0.0111 (8)	
N2	0.4699 (5)	0.1141 (3)	0.1524 (5)	0.0135 (8)	
N3	0.3735 (5)	0.3303 (3)	0.1142 (5)	0.0128 (8)	
H3A	0.3409	0.3580	−0.0116	0.019*	
H3B	0.3911	0.3837	0.2048	0.019*	
H3C	0.2686	0.2873	0.1178	0.019*	
N4	0.8117 (5)	0.3823 (3)	0.2226 (5)	0.0138 (8)	
H4A	0.9371	0.3732	0.3225	0.021*	
H4B	0.7415	0.4358	0.2562	0.021*	
H4C	0.8318	0.3986	0.1058	0.021*	

### Atomic displacement parameters (Å^2^)

**Table:**

	*U*^11^	*U*^22^	*U*^33^	*U*^12^	*U*^13^	*U*^23^
Pt	0.00787 (17)	0.00841 (16)	0.00881 (15)	0.00001 (5)	0.00287 (9)	0.00083 (5)
O2	0.0189 (17)	0.018 (2)	0.0267 (18)	0.0068 (13)	0.0069 (13)	0.0098 (14)
O1	0.0141 (16)	0.030 (2)	0.0294 (18)	0.0024 (14)	0.0119 (13)	0.0097 (16)
O3	0.0252 (17)	0.0128 (18)	0.0188 (16)	−0.0033 (13)	0.0105 (13)	0.0029 (14)
O4	0.0184 (17)	0.030 (2)	0.0145 (16)	−0.0099 (13)	0.0028 (13)	−0.0059 (14)
N1	0.0095 (18)	0.011 (2)	0.0132 (18)	−0.0016 (14)	0.0047 (14)	−0.0013 (15)
N2	0.0148 (19)	0.013 (2)	0.015 (2)	−0.0016 (15)	0.0078 (15)	−0.0025 (16)
N3	0.0111 (18)	0.014 (2)	0.0137 (18)	0.0006 (14)	0.0042 (13)	0.0020 (16)
N4	0.0110 (19)	0.016 (2)	0.0150 (19)	−0.0017 (15)	0.0047 (15)	0.0004 (16)

### Geometric parameters (Å, º)

**Table:**

Pt—N1	1.995 (3)	O4—N2	1.251 (4)
Pt—N2	2.001 (4)	N3—H3A	0.9100
Pt—N3	2.039 (3)	N3—H3B	0.9100
Pt—N4	2.052 (3)	N3—H3C	0.9100
O2—N1	1.233 (4)	N4—H4A	0.9100
O1—N1	1.242 (4)	N4—H4B	0.9100
O3—N2	1.239 (4)	N4—H4C	0.9100
			
N1—Pt—N2	93.06 (13)	Pt—N3—H3A	109.5
N1—Pt—N3	178.67 (13)	Pt—N3—H3B	109.5
N2—Pt—N3	87.85 (13)	H3A—N3—H3B	109.5
N1—Pt—N4	88.58 (13)	Pt—N3—H3C	109.5
N2—Pt—N4	177.96 (13)	H3A—N3—H3C	109.5
N3—Pt—N4	90.53 (14)	H3B—N3—H3C	109.5
O2—N1—O1	118.5 (3)	Pt—N4—H4A	109.5
O2—N1—Pt	122.3 (2)	Pt—N4—H4B	109.5
O1—N1—Pt	119.2 (3)	H4A—N4—H4B	109.5
O3—N2—O4	118.9 (3)	Pt—N4—H4C	109.5
O3—N2—Pt	120.4 (3)	H4A—N4—H4C	109.5
O4—N2—Pt	120.5 (3)	H4B—N4—H4C	109.5

### Hydrogen-bond geometry (Å, º)

**Table:**

*D*—H···*A*	*D*—H	H···*A*	*D*···*A*	*D*—H···*A*
N3—H3*A*···O3^i^	0.91	2.27	3.026 (5)	140
N3—H3*A*···O1^ii^	0.91	2.49	3.107 (5)	126
N3—H3*B*···O4^iii^	0.91	2.22	2.941 (5)	136
N3—H3*C*···O1^iv^	0.91	2.12	3.015 (5)	169
N3—H3*B*···O3^v^	0.91	2.30	2.976 (5)	131
N4—H4*A*···O4^vi^	0.91	2.56	3.392 (4)	153
N4—H4*A*···O1^iii^	0.91	2.57	3.261 (5)	133
N4—H4*B*···O3^v^	0.91	2.14	2.994 (4)	156
N4—H4*C*···O2^i^	0.91	2.18	3.072 (5)	167

Symmetry codes: (i) *x*, −*y*+1/2, *z*−1/2; (ii) *x*−1, −*y*+1/2, *z*−1/2; (iii) *x*, −*y*+1/2, *z*+1/2; (iv) *x*−1, *y*, *z*; (v) −*x*+1, *y*+1/2, −*z*+1/2; (vi) *x*+1, −*y*+1/2, *z*+1/2.
