# Supplementary material for: Crystal structure of cis-diamminebis(nitrito-κN)platinum(II)
Source: Acta Crystallogr E Crystallogr Commun. 2015 Mar 14;71(Pt 4):366–70. doi: 10.1107/S2056989015004879 (PMC4438797; doi:10.1107/S2056989015004879)
Supplement: Supplementary file 3 [file e-71-00366-Isup3.pdf]

Crystal structure of *cis*-diammine-bis(nitrito)-platinum(II)

Volker Kahlenberg,[a]* Thomas Gelbrich,[b] Richard Tessadri[a] and Frederik Klauser[c]

[a]University of Innsbruck, Institute of Mineralogy & Petrography, Innrain 52, A-6020 Innsbruck, Austria, [b]University of Innsbruck, Institute of Pharmacy, Innrain 80, 6020 Innsbruck, Austria, and [c]MED-EL Medical Electronics, Fürstenweg 77a, A-6020 Innsbruck, Austria

Correspondence email: volker.kahlenberg@uibk.ac.at

Supporting information

S1. Hydrogen-bonded structures

Table S1 Geometry for intermolecular hydrogen bonds (Å, °) used for the definition of the HBS (Figure 5b) of *trans*-Pt$(NH_3)_2(NO_2)_2$ (Madarász *et al.*, 2007).

D–H···*A*	*D*–H	H···*A*	*D*···*A*	∠(*D*–H···*A*)
N1–H1···O2 ^{i}	0.90	2.34	3.1479(7)	150
N1–H2···O1^{ii}	0.89	2.25	3.0443(7)	147
N1 –H3···O1^{iii}	0.88	2.49	3.1870(7)	136

Symmetry codes: (i) 1/2–x, –1/2+y, 1–z; (ii) 1/2–x, 1/2+y, 1–z; (iii) 1/2–x, 1/2+y, –z.

Table S2 Geometry for intermolecular hydrogen bonds (Å, °) used for the definition of the HBS (Figure 5b) of *trans*-Pd$(NH_3)_2(NO_2)_2$ (Madarász *et al.*, 2007).

D–H···*A*	*D*–H	H···*A*	*D*···*A*	∠(*D*–H···*A*)
N1–H1···O2^{i}	0.89	2.15	3.0339(6)	170
N1–H2···O2^{ii}	0.89	2.21	3.0846(6)	168
N1 –H3···O1^{iii}	0.89	2.26	3.1361(6)	169

Symmetry codes: (i) x, 1+*y*, *z*; (ii); 1+*x*, 1+*y*, *z* (iii) 1–*x*, –*y*, 1–*z*.

S2. Additional diagrams

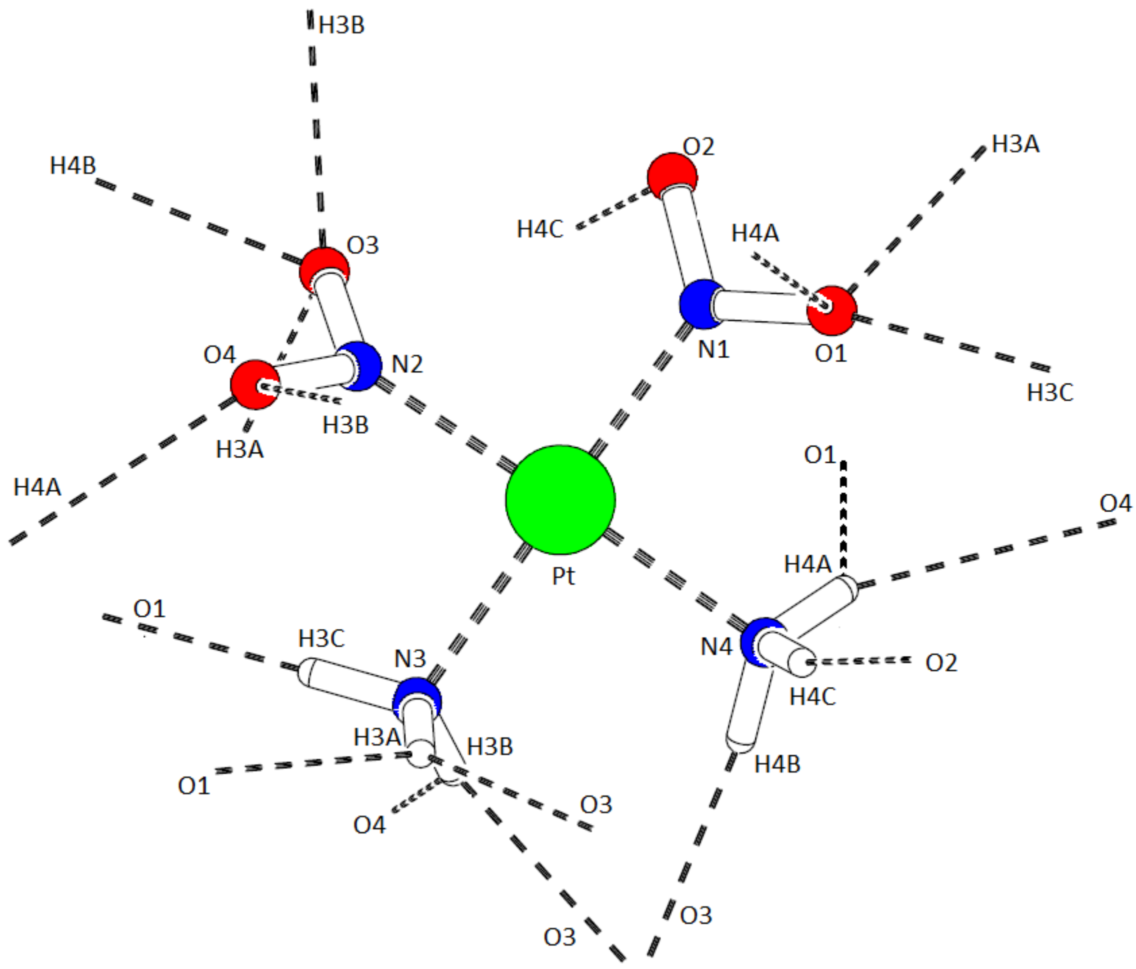

Figure S1 Intermolecular N1–H1···O2 interactions in *cis*-Pt$(NH_3)_2(NO_2)_2$.

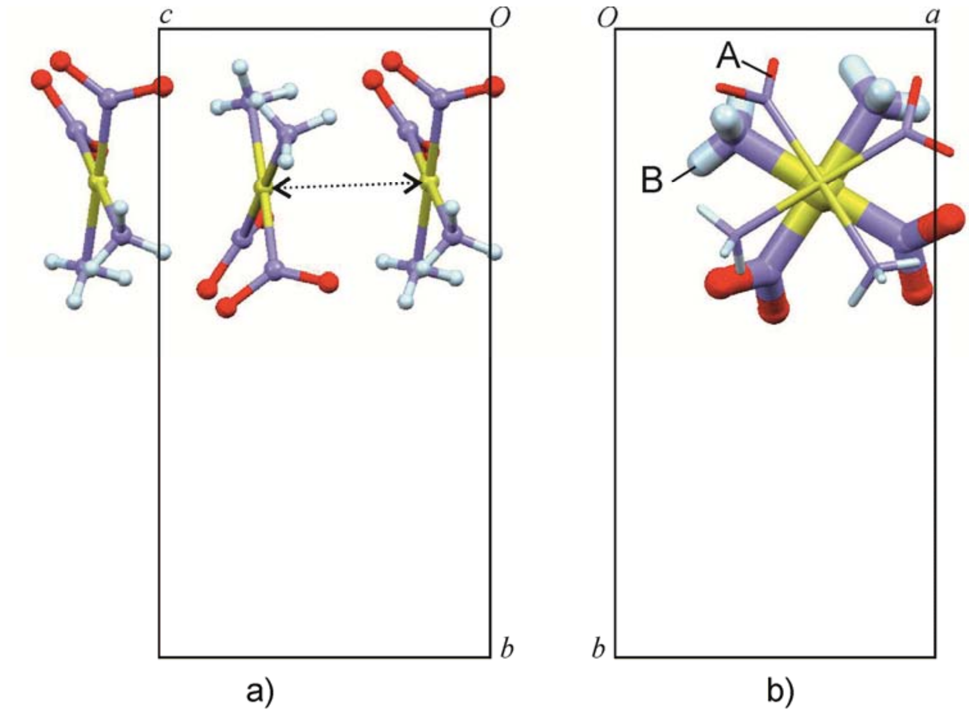

Figure S2 Stack of *cis*-$Pt(NH_3)_2(NO_2)_2$ molecules propagating parallel to the *c* axis: (a) view of a single stack along [100] – the double arrow indicates the shortest Pt···Pt distance (3.549 Å); (b) view of a single stack along [001], showing two neighbouring molecules (denoted A and B) which are related to one another by a glide mirror operation. The two molecules have been drawn in different styles for enhanced clarity.

References

Madarász, J., Bombicz, P., Mátyás, C., Réti, F., Kiss, G. & Pokol, G. (2007). *Thermochim. Acta* **490**, 51–59.
